# Why LATI-H must enter the surgical lexicon

**DOI:** 10.3389/jaws.2026.16806

**Published:** 2026-08-18

**Authors:** Artur Zanellato, James Lucocq, Leo Brown, Thomas M. Drake, Frederik Berrevoet, Stephen J. Wigmore

**Affiliations:** 1 Department of Surgery, Royal Infirmary of Edinburgh, Edinburgh, United Kingdom; 2 Department of General Surgery, Abdominal Wall Reconstruction Unit, The University of Edinburgh, Edinburgh, United Kingdom; 3 Centre for Medical Informatics, Usher Institute, University of Edinburgh, Edinburgh, United Kingdom; 4 Department of General and HPB Surgery and Liver Transplantation, Ghent University Hospital, Ghent, Belgium

**Keywords:** abdominal incisions, caesarean section, hernia surgery, interstitial hernia, intraparietal hernia

## Abstract

**Background:**

Millions of people undergo surgery with utilises a lower abdominal transverse incision (LATI). These incisions offer straightforward surgical access to the abdomen and pelvis and are ubiquitously used worldwide including for caesarean section. The incidence of hernia following these incisions have not been comprehensively studied and there are marked differences in abdominal entry across different LATI types. To address these hernias with a pivotal role in recognition and awareness, we propose a new term for hernias arising from these incisions: Lower Abdominal Transverse Interparietal/Incisional Hernias (LATI-H).

**Methods:**

A narrative review based on relevant literature was conducted to explore how LATI-H is described and reported. We searched the literature using MEDLINE and PubMed from inception to March 2025 to identify relevant studies. We retrieved relevant articles and included articles which described lower abdominal transverse incision complications including their hernias, with no limit on indication. Non-English articles were translated by medical translator for accurate understanding of the technique and literal transcription.

**Results:**

Lower abdominal transverse incisional hernias are poorly described and defined in the literature. They are frequently incorporated to large series of incisional hernias, but they are usually a mix of a large posterior defect with small incisional defects and have different features from other types of incisional hernia. This makes lower abdominal transverse interparietal hernias (LATI-H) a concern for general surgeons due to difficult diagnosis. LATI-H can progress and risk serious complications such as bowel obstruction and bowel ischemia.

**Conclusion:**

Lower abdominal transverse interparietal hernias (LATI-H) are poorly defined and are often erroneously termed Pfannenstiel or C-section hernias. The authors suggest the use of LATI-H to provide clear definition and provide a framework for future research to identify how these arise and find strategies to prevent and repair these hernias effectively.

## Introduction

Lower Abdominal Transverse Interparietal Hernias are a very under studied and under recognized problem. Lower Abdominal Transverse Incisions (LATI) are used by several specialities to provide surgical access to the lower abdomen and pelvis. This includes gastrointestinal, urological, gynaecological and obstetric procedures–most notably caesarean section. Therefore, Lower Abdominal Transverse Incisions (LATI) have major importance as an approach for surgical access for a diverse range of conditions. For childbirth alone in 2015, nearly 30 million are performed per year [1]. However, long-term complications following LATIs are poorly described. For example, the incidence of incisional hernia following caesarean section has been estimated to be between 0% and 5.6% [2], which means that thousands of hernias will occur per year, primarily in young women, and may be overlooked cause of chronic pelvic symptoms worldwide.

The incidence and characteristics of hernia formation following lower abdominal transverse incisions have not been comprehensively investigated. Existing literature primarily focuses on midline or paramedian incisions, leaving a significant gap in understanding hernias associated with lower abdominal transverse approaches. Furthermore, there are notable variations in the technique and anatomical planes of abdominal wall entry among the different types of Lower Abdominal Transverse Incisions (LATI), such as the Pfannenstiel, Maylard, and Cherney incisions. These variations may influence the risk, site, and clinical presentation of postoperative hernias, yet this has not been systematically analysed.

To enhance recognition, classification, and reporting of these unique hernias, we propose a new term: Lower Abdominal Transverse Interparietal Hernias (LATI-H). This nomenclature aims to standardize the identification of hernias arising specifically from LATI approaches and to underscore their distinct anatomical and clinical characteristics. By introducing the LATI-H designation, we aim to promote awareness among surgeons and researchers, facilitate consistent documentation, and lay the foundation for future investigations into their prevention, diagnosis, and optimal management.

## The origin of the term interparietal

### Historical perspective

Interparietal hernias represent an old recognised yet persistently misunderstood entity in abdominal wall surgery. Biomechanically, defects in the posterior abdominal wall permit contents to herniate through and become trapped within the abdominal wall. The history of Lower Abdominal Transverse Incisions and their hernias spans more than three centuries, reflecting the evolution of anatomical knowledge and surgical exploration.

The earliest descriptions of interparietal hernias date back to the 17th century, with observations attributed to early anatomists who noted hernial sacs developing between the layers of the abdominal wall rather than following the expected inguinal canal trajectory [3]. However, it was not until the 18th and 19th centuries that these hernias were more systematically described. Authors such as Petit, Cloquet, and Hesselbach recognised aberrant hernia paths and emphasised the role of abnormal anatomy in preventing normal descent of the hernia sac into the scrotum [4].

The term interparietal hernia gained wider acceptance in the 19th century, particularly through the work of Sir Astley Cooper, who provided detailed anatomical dissections illustrating hernial sacs lodged between the layers of the abdominal wall musculature rather than beneath the skin or into the scrotum [5]. Cooper’s work was pivotal in recognising that these hernias were not surgical curiosities, but rather a consequence of specific anatomical and developmental constraints [6]. was the oldest citation found where the author presented 14 pages of discussion about the “Hernie inguino-interstitielle”.

By the late 19th and early 20th centuries, authors such as [7, 8] further refined classification systems, distinguishing between properitoneal, interstitial, and superficial interparietal hernias. These classifications remain foundational today. Importantly, these early surgeons recognised that many interparietal hernias were diagnosed only intraoperatively or at autopsy, often in the context of bowel obstruction, strangulation, or unexplained groin pain. Mortality rates reported in early series were high, reflecting both delayed diagnosis and limited surgical options of the era.

A recurring historical theme is the strong association between interparietal hernias and congenital anomalies, particularly undescended or ectopic testes. Nevertheless, numerous reports of interparietal hernias in women and in men with normally descended testes challenged a purely congenital explanation and suggested a multifactorial etiology involving mechanical forces, repeated hernia reduction, and acquired weaknesses of the abdominal wall.

Despite several of reported cases throughout the 19th and 20th centuries, interparietal hernias gradually faded from mainstream surgical consciousness [9–24]. As imaging techniques improved and hernia classifications simplified, these complex anatomical variants were often subsumed under broader diagnostic categories or overlooked entirely. This historical neglect has contributed to ongoing underdiagnosis and misattribution of symptoms.

Revisiting the historical literature reveals that interparietal hernias were never truly rare; rather, they were well recognised by earlier surgeons who relied on meticulous anatomical exploration [24, 25].

### The iatrogenic interparietal hernia (lower abdominal transverse interparietal hernia LATI-H)

During the 1800s, the use of lower abdominal transverse incision was uncommon due to concerns about significant haemorrhage and mortality [26], until Johannes Pfannenstiel, described and reported it as a useful incision for caesarean sections in 1900 [27]. His thesis provided evidence that it was a quick, cosmetically sound, and safe approach to the uterus with a significant advantage of low hernia rate. It grew so popular that whenever a lower abdominal transverse scar is discovered during a physical examination, the non-specialist physician will probably characterise and record it as a ‘Pfannenstiel incision’ in the medical notes. However, Pfannenstiel acknowledged that his incision was in fact a modification of an earlier LATI method, “suprasymphyseal cross section” [28] (in which skin is open transversely, but fascia longitudinally) by Otto Ernst Küstner in 1896; a technique which is still used by some in contemporary surgical practice. Few years later Alfred E. Maylard described, in 1907, higher transverse incision where all the layers including rectus muscle were incised transversely [29]. Later, in 1941, Leonid S. Cherney, proposed a modification on Maylard’s technique called “New Transverse Low Abdominal Incision” advocating a much lower incision of the rectus muscle, across its tendon 1 cm from pubic bone [30]. For more than 50 years Pfannenstiel was Joel-Cohen, in 1972, described a higher and straight incision for avoiding bladder injury, opening the peritoneum transversely, not longitudinally [31]. His incision became well recognised by reducing operative time for hysterectomy and later was also used for c-sections. The most recent advancement in non-cutting LATI was in the 1990’s, when Michael Stark modified Joel-Cohen incision. Stark proposed sharp dissection and not closing the peritoneum [32].

LATI are less prone to large incisional hernias and therefore have a low incidence compared with midline incisions. Incidence of hernia in Lower Abdominal Transverse Incisions are reported to be lower compared to midline vertical incisions [24, 33–38]. However, the fact of the abdominal wall is open transversely in layers, is the explanation why the hernia is not full thickness, as most of the time only one layer is compromised causing the interparietal herniation. Interparietal hernias occurring after any type of LATI are usually miscalled Pfannenstiel hernias, but this does not address the physiopathology and specific complications to one or other method. Currently it is impossible to link one of the methods to a specific presentation or complication and therefore a more generic classification would be more appropriate.

### LATI-H complexity

Similar to what was described by Lower and Hicken [25], these hernias are a challenging diagnosis and usually pick up during a surgical procedure. In 1946, Dickinson conducted a review of the literature to report three cases of occult hernia [25], confirming that the symptoms were often confusing and that in most cases diagnosis was made during surgery or occasionally at autopsy, which still happen nowadays [39]. So, having these hernias for a long period causing pain is not uncommon and likely many chronic pain and IBS are related to his pathology.

Computed tomography allows the evaluation of suspected abdominal hernias and detection of clinically occult hernias [40–42] although some ventral hernias may not be apparent when the patient is supine. Dynamic ultrasound is good alternative, but it may lack specificity, and diagnostic laparoscopy may be required for a definitive diagnosis [43]. Modrzejewski reported three cases of interstitial hernia found during laparoscopic exploration of the front abdominal wall performed due to incisional wound site pain where ultrasound was negative [44]. Misinterpretation or misdiagnosis of imaging findings may occur. In our experience, LATI-H have been reported radiologically as divarication, diastasis, intraparietal lipoma, or omentum adherence to fascia as the eyes are usually focused on the herniation through the whole abdominal wall and herniations between layers are very subtle and can be neglected.

## Discussion

The challenge in determining the true prevalence of hernias resulting from LATI is that they are frequently termed incisional [45, 46], intraparietal, interparietal, interstitial hernia [47, 48] or named suprapubic incisional hernias [49, 50] apart of the names Pfannnenstiel or C-section hernias.

Several LATI-H lateral to the EIT ambivium line are usually reported as Spigelian hernias. Even though they look similar in location and in morphology, their physiopathology is completely different.

Spigelian hernias are primary hernias that occurs through the Spigelian fascia, a defect in the aponeurosis of the transversus abdominis and internal oblique muscles, bounded medially by the rectus abdominis and laterally by the linea semilunaris.

Lateral LATI-Hs are also iatrogenic when the incision was extended beyond the lateral border of the rectus sheath (EIT Ambivium linea [51]) and the posterior muscular layers are not closed properly or left open. Therefore they should not be mislabelled as Spigelian but named and classified as LATI-H for better reporting of these defects and future guidelines on the management.

## Future research

Standardisation of terminology used when describing these hernias and where possible identification the incision used are imperative for better understanding of the LATI-H. LATI-H may be a major cause of morbidity following pelvic surgery worldwide, particularly in women. Therefore, it is important there is a defined means to classify hernia types arising from these incisions.

We therefore suggest referring to these hernias as LATI-H (lower abdominal transverse incisional/interparietal hernia). This will subsequently allow for the rational design of clinical trials to understand their aetiology, prevent hernias, and identify optimal surgical treatment strategies.

Better means of defining these hernias provides a foundation to define disease, allowing patients to be recruited to clinical trials and the development of core descriptor/outcome sets. Development of these will provide a rich set of tools to improve outcomes for the overwhelmingly female population who suffer with these hernias, but relies on the definition of disease we propose here, the LATI-H.
